# Effect of acupuncture in the acute phase of intracerebral hemorrhage on the prognosis and serum BDNF: a randomized controlled trial

**DOI:** 10.3389/fnins.2023.1167620

**Published:** 2023-04-13

**Authors:** Lingcong Li, Xiujing Wang, Jiaxun Guo, Yilin Chen, Zhenyu Wang

**Affiliations:** Department of Rehabilitation Medicine, Yongchuan Hospital of Chongqing Medical University, Chongqing, China

**Keywords:** acupuncture, intracerebral hemorrhage, brain-derived neurotrophic factor (BDNF), stroke, prognosis

## Abstract

**Background:**

Intracerebral hemorrhage (ICH) is a common cerebrovascular disease, with a high rate of disability. In the literature on Chinese traditional medicine, there is increasing evidence that acupuncture can help hematoma absorption and improve neurological deficits after cerebral hemorrhage. Brain-derived neurotrophic factor (BDNF), one of the most studied neurotrophic factors, is involved in a variety of neurological functions and plays an important role in brain injury recovery. We investigated the effect of acupuncture intervention in the acute phase of ICH on the prognosis and serum BDNF levels of several patient groups.

**Objective:**

To investigate the influence of acupuncture on the prognosis and brain-derived neurotrophic factor (BDNF) levels in patients in the acute phase of ICH.

**Methods:**

From November 2021 to May 2022, 109 subjects were consecutively enrolled, including patients with ICH, who were randomized into the acupuncture group (AG) and sham acupuncture group (SAG), and a control group (CG). The CG received the same acupuncture intervention as the AG, and the SAG received sham acupuncture, with 14 interventions in each group. The level of consciousness of patients with ICH was assessed and serum BDNF levels were measured in all three groups before the intervention and at 3 weeks after onset, and the level of consciousness and outcomes were assessed at 12 weeks after onset.

**Results:**

After the intervention, the level of consciousness of the AG improved significantly (*P* < 0.05); the BDNF level of only the AG increased significantly (*P* < 0.05); the changes in Glasgow Coma Scale (GCS) score and BDNF level were significantly greater in the AG than in the SAG (*P* < 0.05), especially for locomotion (*P* < 0.05). At 12 weeks post-onset, the AG showed better outcomes and recovery of consciousness than the SAG (*P* < 0.05).

## 1. Introduction

Intracerebral hemorrhage (ICH) refers to the rupture of blood vessels in the brain, resulting in bleeding in the brain parenchyma, ventricles, and subarachnoid space; it accounts for 10–15% of all stroke types (Al-Kawaz et al., 2020).

Brain-derived neurotrophic factor (BDNF) is a peptide growth factor involved in a variety of neurological functions, such as cell growth, differentiation, and plasticity (Mang et al., 2013; Polacchini et al., 2015), thereby affecting cognition, memory, and other functions. It is derived mainly from central nervous system neurons and to a lesser extent from peripheral blood cells, among others (Polacchini et al., 2015). It has been shown that intracranially produced BDNF can cross the blood-brain barrier (Pan et al., 1998); therefore, the level of peripheral BDNF after stroke may reflect central BDNF levels. After stroke onset, BDNF can enhance local anti-inflammatory action by upregulating IL-10 and downregulating TNF-α (Jiang et al., 2010) expression.

In Chinese medical theory, intracerebral hemorrhage (known as Zhongfeng in Chinese medicine) is based on an internal deficiency of Qi (the potential energy that maintains your life and every body system) and blood, and is triggered by exertion, worry and poor diet, resulting in an imbalance of internal Yin and Yang, and thus cerebral vascular blockage or blood overflow. Therefore, in terms of treatment, it is important to differentiate according to Yin and Yang so as to “treat the same disease differently” or “treat different diseases with the same treatment.” Acupuncture is an important treatment tool, and several previous studies (Fan and Liu, 1997) have found that acupuncture in the acute phase of intracerebral hemorrhage has better outcomes than in other phases. Acupuncture is well accepted in China as an alternative and complementary strategy after stroke, as recommended by the World Health Organization (Lehmann, 2013). Some studies have suggested that acupuncture can improve post-stroke dysfunction through a variety of mechanisms, including anti-apoptosis in ischemic areas (Chavez et al., 2017). A randomized controlled trial found that acupuncture reversed the downregulation of serum BDNF (Jiang et al., 2018). Xiong et al. (2020) found that head acupuncture combined with cognitive function training resulted in a pronounced increase in BDNF levels and improved post-stroke outcomes. These findings suggest that one of the mechanisms by which acupuncture improves functional impairment after stroke may involve serum BDNF levels.

Regarding the timing of rehabilitation interventions, overseas recommendations suggest starting rehabilitation within 24–48 h after the onset of stroke (Greenberg et al., 2022), whereas Chinese guidelines usually suggest starting rehabilitation 48 h to 1 week after the onset of stroke or after the stabilization of vital signs (Yu et al., 2020). The Xingnao-Kaiqiao acupuncture method is commonly used in clinical practice to treat stroke. It has been known to revive the human brain, and can improve the hypoxic state of brain tissue, leading to functional recovery (Li and Han, 2015).

In this trial, acupuncture was applied starting from the acute phase [generally considered to be 2–7 days after onset (Manning et al., 2014)] of ICH, and the participants were divided into acupuncture (AG), sham acupuncture (SAG), and control groups (CG) to compare BDNF levels in each group. The relationship between acupuncture and BDNF was investigated by comparing the changes in BDNF levels in each group and by analyzing the outcome between the groups. The efficacy of acupuncture for outcomes of ICH was determined in order to provide a reference for acupuncture treatment protocols in the acute phase of ICH.

## 2. Subjects and methods

### 2.1. Trial design

This double-blind, randomized controlled trial was conducted in accordance with the Declaration of Helsinki and approved by the Ethics Committee of Yongchuan Hospital of Chongqing Medical University (No. 110, 2021). This study was registered at ClinicalTrials.org (NCT05479903). Patients with ICH who met the inclusion criteria were randomly divided into the AG or SAG using the SAS software (version 9.4). All patients with ICH underwent poststroke secondary prevention, treatment of underlying diseases, and comprehensive rehabilitation training. Accordingly, the AG was administered an acupuncture intervention and the SAG was administered sham acupuncture intervention. When the medical condition changed and the intervention was no longer appropriate, it was suspended; it was resumed after the condition had stabilized. Fourteen interventions were performed in all groups, with the first intervention administered no longer than 7 days after onset and the last intervention administered no longer than 3 weeks after onset. The CG was administered the same intervention protocol as the AG. Serum BDNF levels were measured in all three groups before and after the interventions, changes in patient consciousness were assessed using the Glasgow Coma Scale (GCS), and patient outcomes were assessed using the modified Rankin Scale (mRS) and GCS at week 12 after onset. All included patients or their guardians signed an informed consent form before entering the trial.

### 2.2. Participants

From November 2021 to May 2022, patients with ICH were consecutively enrolled from the Department of Neurosurgery or Department of Rehabilitation Medicine of Yongchuan Hospital of Chongqing Medical University. Patients who visited the physical examination center during the same period were enrolled to the CG.

#### 2.2.1. ICH inclusion criteria

The incision criteria for the AG and SAG were as follows: (1) age ≥ 18 years, (2) confirmed lesion on brain CT or MRI, (3) mRS ≤ 1 before onset, (4) time between symptom recognition and admission ≤ 72 h, (5) diagnosis of intracerebral hemorrhage by two regular physicians according to the Chinese Guidelines for the Diagnosis and Treatment of Cerebral Hemorrhage (2019), and (6) stable vital signs within 7 days after onset. The inclusion criteria for the CG were (1) age ≥ 50 years; (2) mRS ≤ 1; and (3) normal cognitive, motor, and speech function.

#### 2.2.2. Exclusion criteria for all subjects

The exclusion criteria were as follows: (1) History of intracranial surgery or trauma, (2) neurological or psychiatric disease, (3) stroke; (4) abnormal coagulation function; (5) physical disability prior to onset; and (6) participation in other clinical trials within the previous 3 months.

#### 2.2.3. Dropping out

Subjects with worsening medical conditions or serious complications that made them unfit for continuing the trial, subjects with poor compliance who failed to complete the 14 interventions within 3 weeks of onset, and subjects who discontinued participation for personal reasons were dropped from the analysis, and the reasons for withdrawal and dropping out were recorded.

### 2.3. Intervention

The basic disease treatment for AG and SAG, secondary prevention interventions after stroke, and comprehensive rehabilitation training were carried out with reference to relevant guidelines (Zhang et al., 2019; Al-Kawaz et al., 2020). The AG and CG were administered the Xingnao-Kaiqiao Acupuncture Method; the SAG was administered sham acupuncture.

#### 2.3.1. Selection of acupoints

Neiguan (PC06), Shuigou (GV26), and Sanyinjiao (SP06) were the main acupoints, with Jiquan (HT01), Weizhong (BL40), and Chize (LU05) on the affected side as the secondary acupoints. All the acupoints were selected and positioned according to GB/T123462006 (Gb/T12346-2006, 2006), and the manipulation techniques refer to the research (Shi, 2005) of Academician Shi Xuemin.

#### 2.3.2. Intervention steps

The subject was placed in a supine position, and the acupuncture operator disinfected both hands and the skin at the acupoint using povidone iodine solution (concentration 5%, Chongqing Xieran Pharmaceutical Co., Ltd., China). Then, a disposable electrode piece (LHJ-5680, Chongqing Younaite Medical Equipment Co., Ltd., China) was used to cover the skin at the selected acupoint, with or without piercing the electrode piece with disposable acupuncture needles (0.30 × 50 mm, Suzhou Dongbang Medical Devices Co., China). The location and manipulation of the AG and CG acupoints are shown in Table 1. Subjects suitable for acupuncture were given the intervention once a day for 30 min each.

**TABLE 1:** Acupoints selected in the study.

	Location	Manipulation
Neiguan (PC06)	On the forearm: 6.5 cm above the transverse wrist palmaris, between the palmaris longus tendon and the radial carpal flexor tendon	Vertical insertion at a depth of 10–15 mm applied with the twisting, lifting, and thrusting technique of the reducing method^1^ for 1 min.
Shuigou (GV26)	On the face: median depression above the upper lip	Oblique insertion at a depth of 5–10 mm toward the nasal septum; heavy bird-pecking needling^2^ is applied until tear formation is observed in the patient’s eyes.
Sanyinjiao (SP06)	On the crus: 10 cm directly above the tip of the medial malleolus, posterior to the medial border of the tibia	Inserted obliquely along the inner edge of the tibia at an angle of 45° to the skin to a depth of 10–15 mm and applied with the twisting technique of the reinforcing method^3^; three twitches of the lower limbs are considered appropriate.
Jiquan (HT01)	Apex of the armpit	Insert vertically 15–20 mm next to the Jiuquan at a depth of 10–15 mm, using the twist, lift and reducing method,^4^ with three upper limb twitches as appropriate.
Weizhong (BL40)	Midpoint of the transverse popliteal line	Straight leg elevation of the affected limb, vertical insertion at a depth of 5–10 mm; applied with the twisting, lifting, and reducing method^4^; three twitches of the lower limbs are considered appropriate.
Chize (LU05)	Intersection of the radial recess of the biceps tendon with the transverse elbow stripe	Elbow is bent at an angle of 120° and stabbed straight to a depth of 10–15 mm; applied with the twisting, lifting, and reducing method^4^; three twitches of the forearm and fingers are considered appropriate.

^1^The thumb, middle, and index fingers hold the needle handle and twist it back and forth in a forward and backward rotation, then release the needle handle, lift it slightly when it returns to its natural position and then pierce it vertically.

^2^The needle handle is rotated 360 degrees and the needle is then lifted or inserted into the acupuncture point in small, rapid movements, similar to a sparrow pecking at rice.

^3^The needles are lifted slowly, but not completely out of the acupuncture point, and then inserted quickly and forcefully into the point.

^4^The needles are lifted quickly, but not completely withdrawn, and then slowly and gently inserted into the acupuncture point.

The SAG received sham acupuncture intervention using the same procedure and equipment as the AG, with the difference that the acupuncture needle did not pierce the electrode piece, that is, it did not pierce the skin, and the needle was fixed to the electrode piece.

#### 2.3.3. Intervention personnel

**FIGURE 1:**
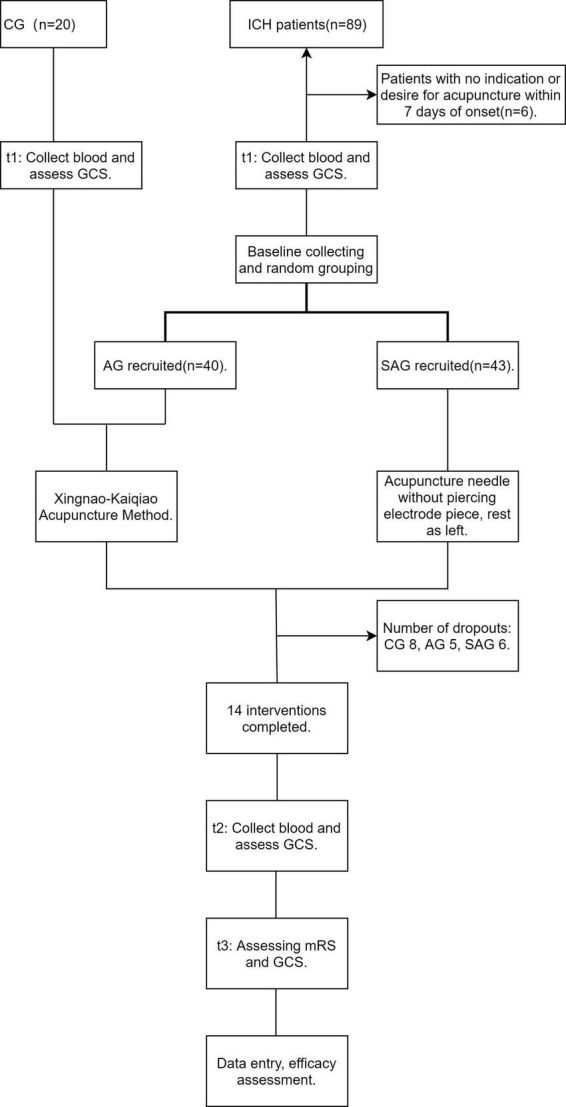
Flow chart of subjects.

All of the acupuncture therapists have completed a relevant medical course, graduated in the field of acupuncture and have obtained a national qualification. They had all been working for more than 5 years and were trained in both the Xingnao-Kaiqiao acupuncture and the sham acupuncture method before the intervention to ensure that each therapist was able to perform the intervention correctly. If bleeding occurred at the acupoints during the intervention, pressure was applied to the points with a sterile cotton swab until the bleeding stopped. If the patient’s condition changed (fever, epilepsy, etc.) such that the intervention was no longer appropriate, the intervention was suspended until the condition had stabilized (Figure 1).

### 2.4. Follow-up and outcomes

#### 2.4.1. Time points for follow-up

The day of onset was defined as d1, the first follow-up (pre-intervention, t1) and the first intervention were completed at d1–d7, the last intervention was completed at d14–d20, the second follow-up (3 weeks after onset, t2) was completed at d20–d22, and the third follow-up (12 weeks after onset, t3) was completed at d82–d86. As far as possible, the intervention and follow-up were carried out with all patients at similar time points of the disease course, and all interventions were completed at t2 and t3 follow-up visits.

#### 2.4.2. Clinical follow-up

Relevant training is provided to all assessors. The assessment of one subject was carried out by the same assessor.

The main outcome indicators were the change in the GCS score and the mRS score. The GCS assesses the patient’s ability to open their eyes and their responses to verbal and motor tasks on a scale of 3–15, with higher scores representing a better state of consciousness. The mRS assesses the ability to perform daily activities on a scale of 0–6, with higher scores indicating poorer ability to take care of oneself. In this trial, mRS scores of 0–2 were defined as “good outcome” and mRS scores of 3–6 were defined as “poor outcome” according to the American Stroke Association guidelines (Lin et al., 2021).

Secondary outcome indicators were changes in BDNF levels and each GCS subcategory. Blood samples were collected from all subjects at t1 and t2. Venous blood (2 mL) was drawn and transferred into tubes containing procoagulant (procoagulant 5 mL, Weihai Weigao Medical Devices Ltd., Weihai, Shandong, China), shaken gently, and allowed to coagulate at room temperature for at least 30 min. Serum was then collected by centrifugation (Neofuge15R, Heal Force Development Ltd.) at 3,000 rpm for 10 min and stored at −80°C until assay. Serum BDNF levels were measured using an ELISA kit (R&D Systems, Inc., USA) by experienced laboratory staff who were blinded to the rest of the trial, and laboratory results were not known to the assessors until the end of the study.

### 2.5. Sample size

This study was a randomized controlled trial, and the sample size was estimated by comparing the means of the two samples, where n1 and n2 are the required numbers of the two samples, δ is the difference between the means of the two groups, and σ is the overall standard deviation: α = 0.05 and β = 0.20. After reviewing the relevant literature (Liu et al., 2016), we set δ/σ = 0.77. Using the tables of two-side tolerance factors, we confirmed that μα = 1.96 and μβ = 0.84, and substituted these into the formula to obtain n≈28, meaning that 28 cases were needed in each group. Considering withdrawals during the trial, the sample size was increased by 20% so that at least 34 subjects needed to be included in each group.


n=1n=22[μα+μβδ/σ]2+14μα2

### 2.6. Randomization

Random numbers were generated by an investigator who was not involved in other parts of the trial using SAS 9.4, and all numbers were assigned to the groups according to prespecified rules. The grouping results were stored in a sealed opaque envelope with the generated order written on its surface. The allocation results were kept by another investigator (Chen) who was not involved in the rest of the trial; when a subject was enrolled, Chen allocated the grouping result according to the generated order and informed the acupuncture operator.

### 2.7. Allocation and blinding

Only the necessary medical staff will be on site during the intervention to reduce the possibility of blind failure of the patients’ families. Until the end of the trial, only Chen and the acupuncture operators were aware of the allocation results; the subjects, assessors, and other investigators were not informed of the grouping results.

### 2.8. Statistical methods

All statistical analyses were performed using Statistical Package for the Social Sciences (SPSS) 26.0 (Chicago, Armonk, NY, USA). The data were assessed for normality using the Kolmogorov–Smirnov test. Measurement data were expressed as median (interquartile range) or mean ± standard deviation, and categorical, as number (rate).

Comparisons between two groups of normally distributed count data were made using the Student *t*-test and among three groups using one-way ANOVA; comparisons between two groups of non-normally distributed count data were made using the Mann–Whitney U test and among three groups using the Kruskal–Wallis test. Multiple time points of count data were compared using the Mauchly’s test of sphericity and Two-way Repeated Measures ANOVA.

The Pearson or fisher chi-square test was used for comparisons of unordered categorical information, the Mann-Whitney U test for comparisons of two groups of ordered categorical information, the Kruskal–wallis for comparisons of three groups of ordered categorical information, and the Pearson chi-square test for cross-tabulations when comparing inter-group outcomes before and after the intervention.

The GCS has three sub-categories with full marks of 4, 5, and 6. To compare the changes in scores between subscales, the absolute value of a given change was divided by the full score of that subscale. For example, the absolute value of the change in eye opening was divided by 4. Statistical analyses were performed as described previously. All results were considered statistically significant at *P* < 0.05.

### 2.9. Reasons for dropped out and adverse reactions

Adverse effects: pain, 20 in CG, 2 in AG and 1 in SAG reported that the intervention caused pain, but all disappeared within 1 h of the end of the intervention. Bleeding, 6 in CG, 19 in AG and 0 in SAG had bleeding at the end of the intervention at the pinpoint, which stopped within 10 min after applying sterile swab pressure. Some ICH patients are unconscious for part or all of the intervention, so that their feelings are not known. No other adverse effects were observed during this trial.

Subjects dropped out for the following reasons: pain, CG = 7; personal reasons, CG = 1, AG = 3, and SAG = 2; failure to complete the 14 interventions on time, AG = 2 and SAG = 4.

## 3. Results

### 3.1. Overall baseline characteristics

Overall, 84 subjects finally completed the full trial and were included in the analysis; the basic information is shown in Table 2. There were no statistically significant differences in any basic information between the 84 subjects who completed the trial and the 109 enrolled subjects (*P* > 0.05). There were no statistically significant differences in gender, age, comorbidities, lifestyle habits, or baseline BDNF levels among the AG, SAG, and CG (*P* > 0.05). There were no statistically significant differences in time from symptom recognition to admission, proportion treated surgically, admission systolic blood pressure, or pre-intervention GCS score between AG and SAG (*P* > 0.05). There was a statistically significant difference in systolic blood pressure between CG and ICH patients on admission (*p* < 0.05).

**TABLE 2:** Baseline characteristics.

	AG (*n* = 35)	SAG (*n* = 37)	CG (*n* = 12)	*P*-value
Female [*n* (%)]	14 (40.0)	15 (40.5)	5 (41.7)	>0.05
Age, years, mean ± standard deviation	58.2 ± 12.7	60.6 ± 11.2	60.8 ± 6.8	>0.05
Cardiac disease [*n* (%)]	5 (14.3)	3 (8.1)	2 (16.7)	>0.05
Hypertension [*n* (%)]	29 (82.9)	29 (78.4)	3 (25.0)	0.000
Diabetes [*n* (%)]	6 (17.1)	4 (10.8)	2 (16.7)	>0.05
Smoking [*n* (%)]	19 (54.3)	16 (43.2)	6 (50.0)	>0.05
Drinking [*n* (%)]	15 (42.9)	16 (43.2)	5 (41.7)	>0.05
Time from symptom recognition to admission, hours, median (interquartile range)	8.0 (5.0–3.0)	6.0 (4.0–17.0)	/	>0.05
Surgical treatment [*N* (%)]	23 (65.7)	26 (70.3)	/	>0.05
Systolic blood pressure at admission >180 mmHg [*n* (%)]	16 (45.7)	15 (40.5)	0 (0.0)	
Systolic blood pressure at admission 160–180 mmHg [*n* (%)]	5 (14.3)	9 (24.3)	2 (16.7)	0.001
Systolic blood pressure at admission 140–159 mmHg [*n* (%)]	8 (22.9)	5 (13.5)	1 (8.3)	
Systolic blood pressure at admission <140 mmHg [*n* (%)]	6 (17.1)	8 (21.6)	9 (75)	
Baseline BDNF levels, ng/mL, median (interquartile range)	24.5 (17.8–30.2)	21.6 (13.8–31.2)	21.7 (17.3–24.3)	>0.05
GCS score pre-intervention, median (interquartile range)	10.0 (8.0–11.5)	9.0 (7.0–12.0)	/	>0.05

BDNF, brain-derived neurotrophic factor.

### 3.2. BDNF before and after the intervention

Table 3 demonstrates the levels of BDNF before and after the intervention (t1 and t2). As shown in the table, only the BDNF levels of AG were significantly higher after the intervention compared to before (*P* < 0.05), while the BDNF levels of SAG and CG were not statistically significantly changed compared to before the intervention (*P* > 0.05). In terms of BDNF change values, AG was more significantly elevated than CG or SAG (*P* < 0.01).

**TABLE 3:** BDNF levels before and after intervention.

BDNF levels, ng/ml, median (interquartile range)	t1	t2	*P*-value
AG	24.5 (17.8∼30.2)	28.0 (26.2∼37.1)	0.035
SAG	21.6 (13.8∼31.2)	19.4 (15.0∼26.3)*	>0.05
CG	21.7 (17.3∼24.3)	22.5 (15.8∼26.7)*	>0.05

*BDNF change values (from t1 to t2) were statistically different from those of AG, p < 0.01.

BDNF, brain-derived neurotrophic factor.

### 3.3. GCS before and after the intervention

The Mauchly’s test of sphericity *P* = 0.003. Using two-way Repeated Measures ANOVA, it was suggested that GCS rose over time in both AG and SAG (*P* < 0.001), but even though both increased over time, the rise in GCS was significantly higher in AG than in SAG (*P* < 0.01). As shown in Table 4, baseline GCS was found to be comparable between AG and SAG (*P* > 0.05). Both at the end of the intervention and 12 weeks after onset, GCS was significantly higher in AG than in SAG (*P* < 0.05).

**TABLE 4:** GCS before and after intervention.

GCS, median (interquartile range)	t1	t2	t3	*P*-value
AG	10.0 (8.0∼11.5)	12.0 (10.0∼15.0)*	15.0 (13.0∼15.0)*	0.000
SAG	9.0 (7.0∼12.0)	11.0 (8.0∼13.0)*	12.0 (10.0∼15.0)*	0.000
*P*-value	>0.05	0.04	0.01	/

*Statistically different compared to GCS at t1 within the group.

GCS, Glasgow Coma Scale.

### 3.4. Change in GCS subscale values and outcomes 12 weeks after onset

Table 5 suggests that the outcome evaluation of AG and SAG using the mRS at 12 weeks after onset was better for AG than for SAG (*p* < 0.05). Of the three aspects evaluated by the GCS, only motor aspects were better recovered in AG than in SAG (*p* < 0.05), while there was no statistical difference in the degree of improvement in eye opening and verbal response between the two (*p* > 0.05).

**TABLE 5:** Change from t1 to t3 in GCS subscale values and outcomes in t3.

	AG (*n* = 35)	SAG (*n* = 37)	*P*-value
Good outcome (mRS ≤ 2) [*n* (%)]	18 (51.4)	9 (24.3)	0.018*
ΔGCS-E/4 (eye opening)	0.3 (0.0∼0.3)	0.0 (0.0∼0.3)	0.212
ΔGCS-V/5 (verbal response)	0.2 (0.2∼0.5)	0.2 (0.0∼0.4)	0.453
ΔGCS-M/6 (motor response)	0.3 (0.2∼0.5)	0.2 (0.0∼0.3)	0.016

*The *P*-value was obtained by Pearson χ^2^ test, χ^2^ = 5.638.

## 4. Discussion

To the best of our knowledge, this study is one of the rare studies to focus on the efficacy of acupuncture in the acute phase of ICH, which is characterized by high mortality and disability as a subtype of stroke. In designing the trial, we simultaneously explored changes in BDNF levels after the intervention and ICH outcomes, while adjusting for confounding factors such as natural disease progression and physiological effects of acupuncture in healthy humans by employing the SAG and CG.

For the timing of needle intervention in patients with cerebral hemorrhage, previous guidelines (Lin et al., 2019) or meta-analyses (Liu et al., 2017) have recommended intervention in the acute phase, but some clinicians have abandoned needle treatment in the acute phase to prevent coagulation disorders in patients after invasive stimulation. There were no severe adverse effects of acupuncture in this trial, and none of the subjects withdrew owing to dissatisfaction with the efficacy, suggesting that the safety of acupuncture is reliable. Our study suggests that acupuncture shows positive effects in both short-term recovery of consciousness and long-term improvement in quality of life. In clinical practice, acupuncture, which has the advantages of being inexpensive and available at home, is less difficult to promote, and Xingnao-Kaiqiao acupuncture in particular may bring significant benefits to patients.

We also found that the CG had a 40% dropout rate, with seven of the eight dropouts withdrawing because of pain. To ensure a control effect, we administered the same interventions to the CG and AG, that is, Xingnao-Kaiqiao acupuncture. The majority of patients in the AG were in a coma, and this intervention was helpful in promoting awakening, but the strength of the intervention was too strong for conscious people to tolerate; therefore, only 12 people in the CG completed the full intervention, which in turn resulted in a small number of CG subjects and may have increased the sample bias. In the future, if similar trials are conducted, the high dropout rate of the CG due to pain will need to be addressed or more CGs need to be included.

Over half of the patients were unconscious during the intervention, which improved the blinding reliability of the interventions. However, it is worth noting that although the SAG was not affected by the direct penetration of the needles into the skin, this does not mean that sham needling would not have a therapeutic effect. Chae et al. (2018) argued that sham needling could have some efficacy, as it generates tactile sensation. After the end of all interventions, consciousness improved in both groups of patients with ICH compared to the pre-intervention levels, indicating that there may be other mechanisms of recovery from ICH besides the effect of acupuncture. The recovery of consciousness and the magnitude of BDNF changes were more pronounced in the AG than in the SAG, suggesting that acupuncture likely helped patients recover more quickly by enhancing mechanisms related to the production of BDNF. Among the three dimensions of the GCS assessment, we found greater improvements in motor response in the AG than in the SAG, suggesting a specific modulatory effect of acupuncture on the motor-related network of stroke patients (Zhang et al., 2021). In addition to this, Zhang et al. (2021) suggested that acupuncture promotes the recovery of verbal functions by regulating neuroplasticity, which can help patients return to society as soon as possible. After the intervention, only the BDNF level of the AG changed significantly after the intervention, suggesting that there may not be a significant effect of sham acupuncture or acupuncture directed to BDNF levels in healthy bodies. In other words, the specific changes that occurs after ICH may be a prerequisite for acupuncture to have an effect on BDNF levels.

This study has some limitations. First, as a product of secretion, peripheral levels of BDNF may be influenced by drugs, and although the effect of interfering factors could be reduced in the AG in comparison with the SAG, we did not restrict patient drug intake during basal treatment, and it is unclear whether changes in BDNF levels in the AG were caused by specific drugs or other unknown factors. Second, this was a single-center trial, and the number of subjects who completed the full trial was 84; therefore, selection bias is inevitable. Third, although we trained each acupuncture operator, differences in techniques between operators may have led to individual differences in efficacy. Fourth, previous studies have suggested that changes in BDNF levels differ at different stages after stroke (Di Lazzaro et al., 2007; Jimenez et al., 2009; Chan et al., 2015; Zhang et al., 2017), and although we controlled the analysis for time from symptom recognition to admission and time from enrolment to onset, we may still have missed the observation time window for BDNF changes in some patients.

## 5. Conclusion

Acupuncture in the acute phase of ICH has a significant positive effect on both short-term recovery of consciousness and long-term outcomes in patients with ICH, and the production of BDNF may be associated with this effect.

## Data availability statement

The raw data supporting the conclusions of this article will be made available by the authors, without undue reservation.

## Ethics statement

The studies involving human participants were reviewed and approved by the Ethics Committee of Yongchuan Hospital of Chongqing Medical University. The patients/participants provided their written informed consent to participate in this study.

## Author contributions

ZW designed the trial protocol. YC kept the grouping results until the end of the trial. XW and JG recruited and followed the subjects. LL was responsible for analyzing the data and writing the manuscript. All authors contributed to the article and approved the submitted version.
